# Supplementary material for: New Propargyloxy Derivatives of Galangin, Kaempferol and Fisetin—Synthesis, Spectroscopic Analysis and In Vitro Anticancer Activity on Head and Neck Cancer Cells
Source: Cells. 2023 Sep 15;12(18):2288. doi: 10.3390/cells12182288 (PMC10528839; doi:10.3390/cells12182288)
Supplement: Supplementary file 1 [file cells-12-02288-s001.zip › cells-2592073-supplementary.pdf]

cells

MDPI

Supplementary material

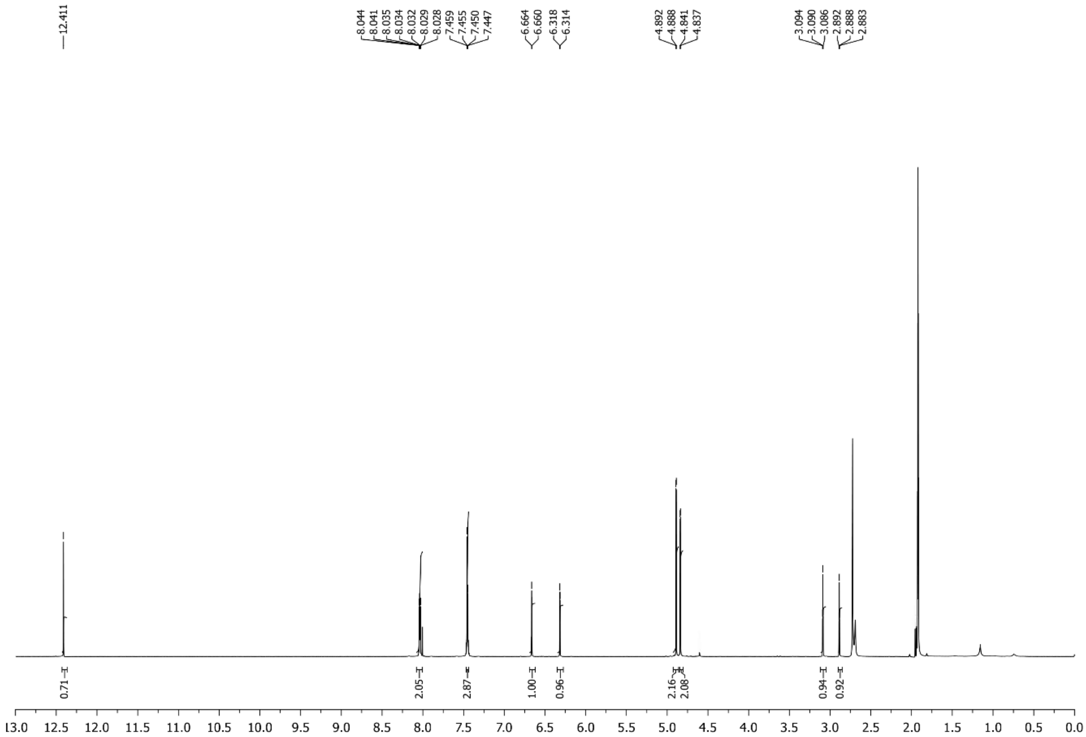

Figure S1. The ^{1}H NMR spectrum (600 MHz, aceton-d6) of 5-hydroxy-2-phenyl-3,7-bis(prop-2-yn-1-yloxy)- 4H-chromen-4-one **4**.

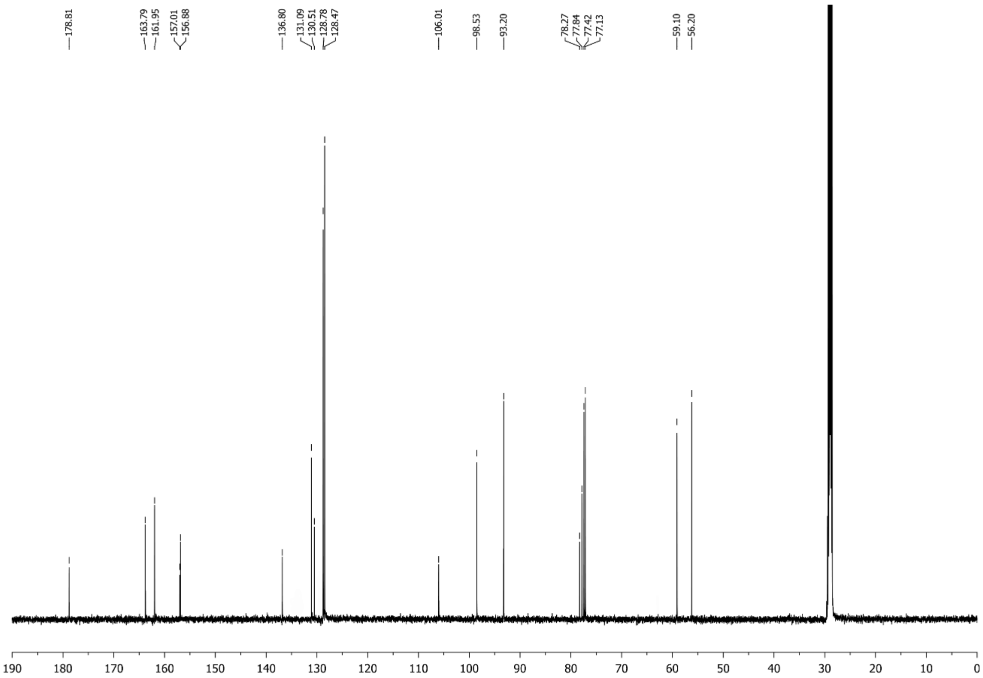

Figure S2. The ^{13}C NMR spectrum (150 MHz, aceton-d6) of 5-hydroxy-2-phenyl-3,7-bis(prop-2-yn-1-yloxy)- 4H-chromen-4-one **4**.

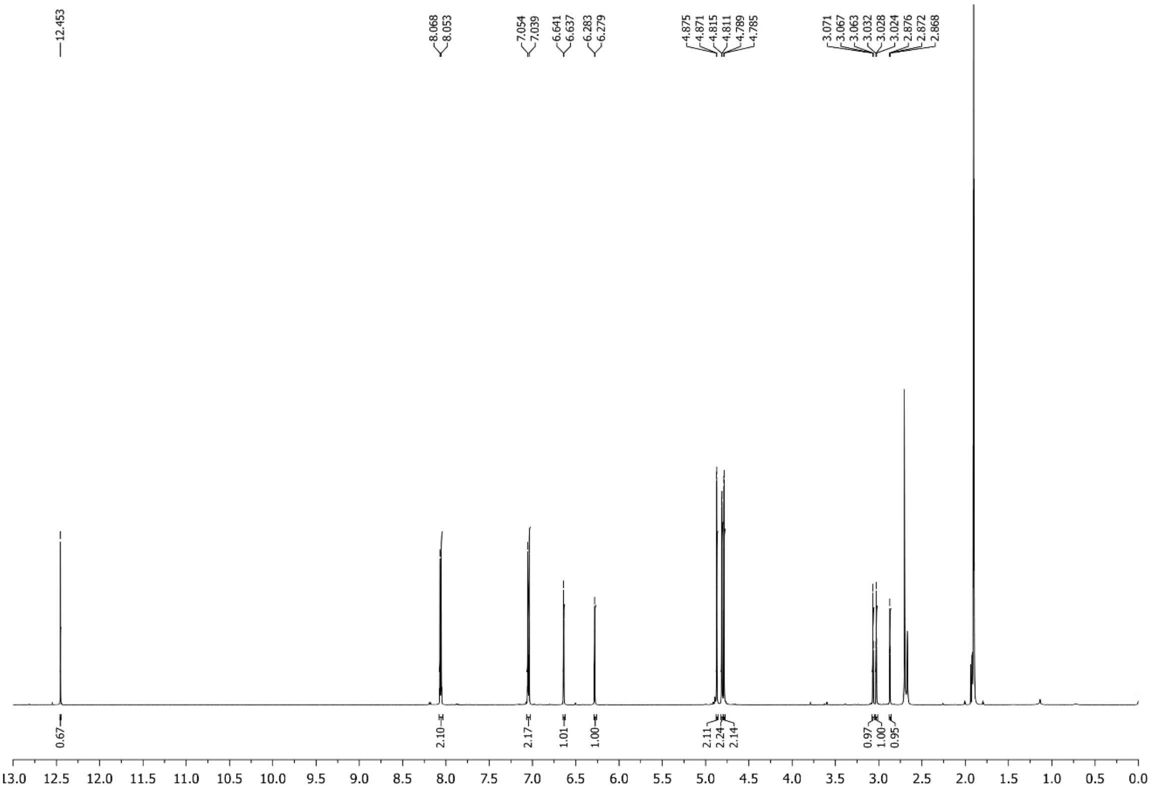

Figure S3. The ^{1}H NMR spectrum (600 MHz, aceton-d6) of 5-hydroxy-3,7-bis(prop-2-yn-1-yloxy)-2-(4-(prop-2-yn-1-yloxy)phenyl)-4H-chromen-4-one **5**.

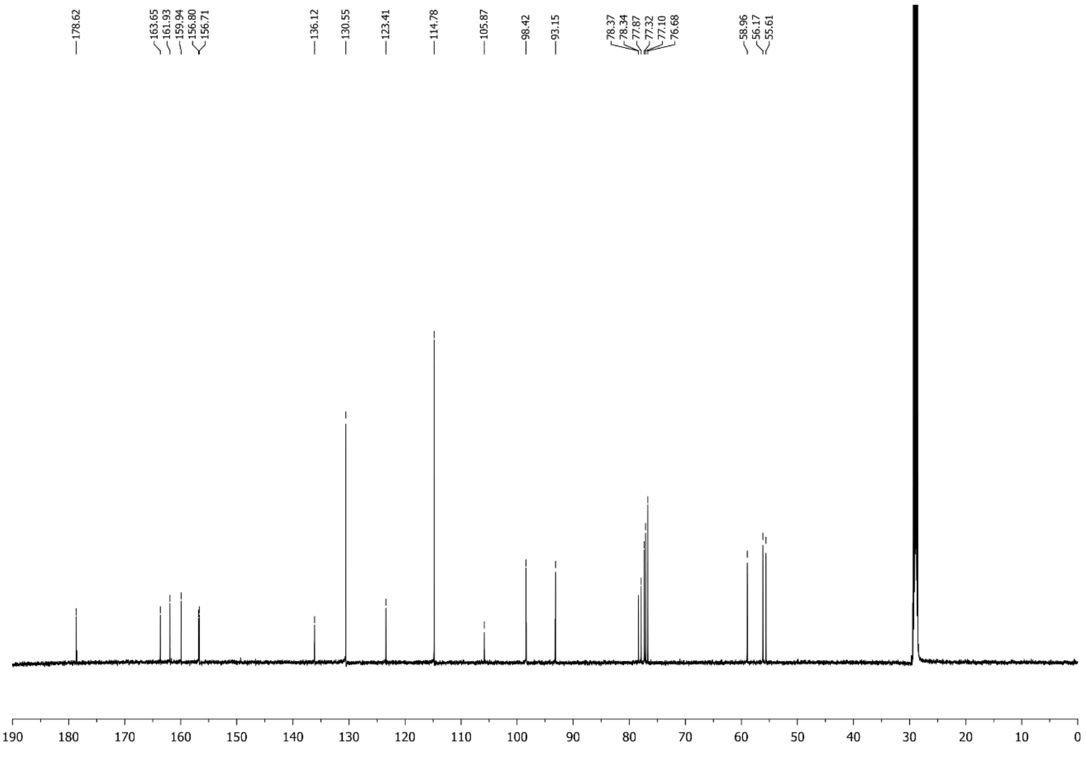

Figure S4. The ^{13}C NMR spectrum (150 MHz, aceton-d6) of 5-hydroxy-3,7-bis(prop-2-yn-1-yloxy)-2-(4-(prop-2-yn-1-yloxy)phenyl)-4H-chromen-4-one **5**.

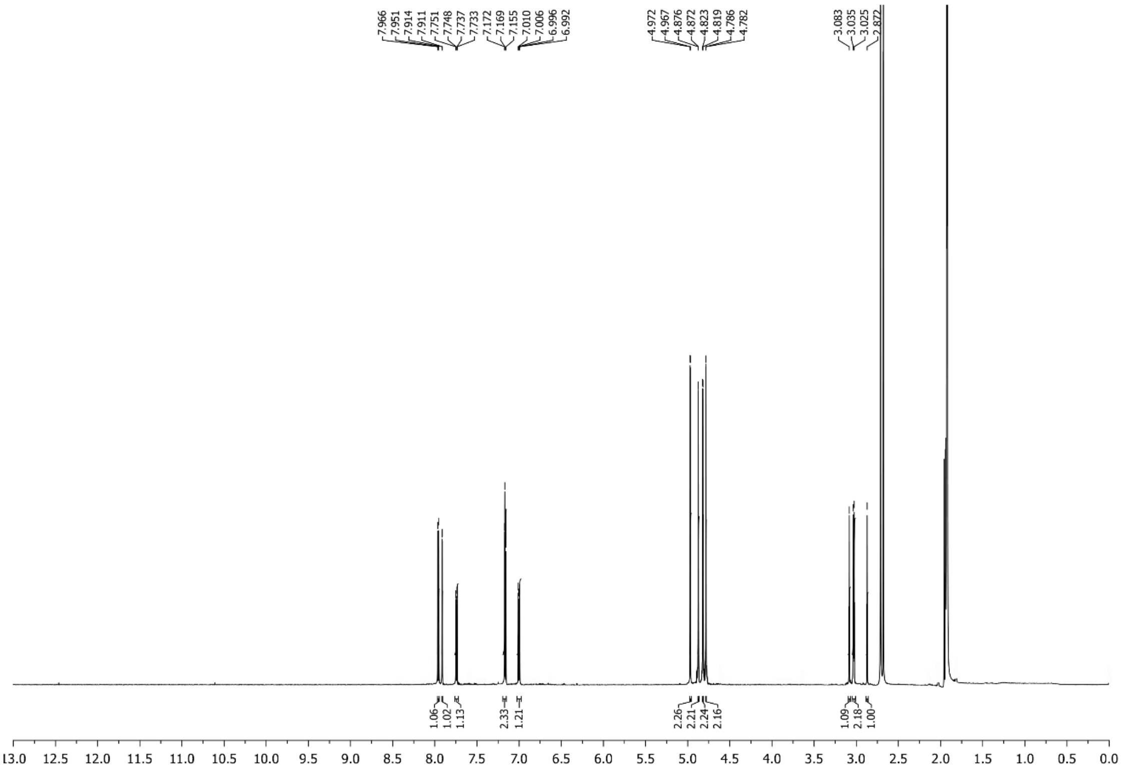

Figure S5. The ^{1}H NMR spectrum (600 MHz, aceton-d6) of 2-(3,4-bis(prop-2-yn-1-yloxy)phenyl)-3,7-bis(prop-2-yn-1-yloxy)- 4H-chromen-4-one **6**.

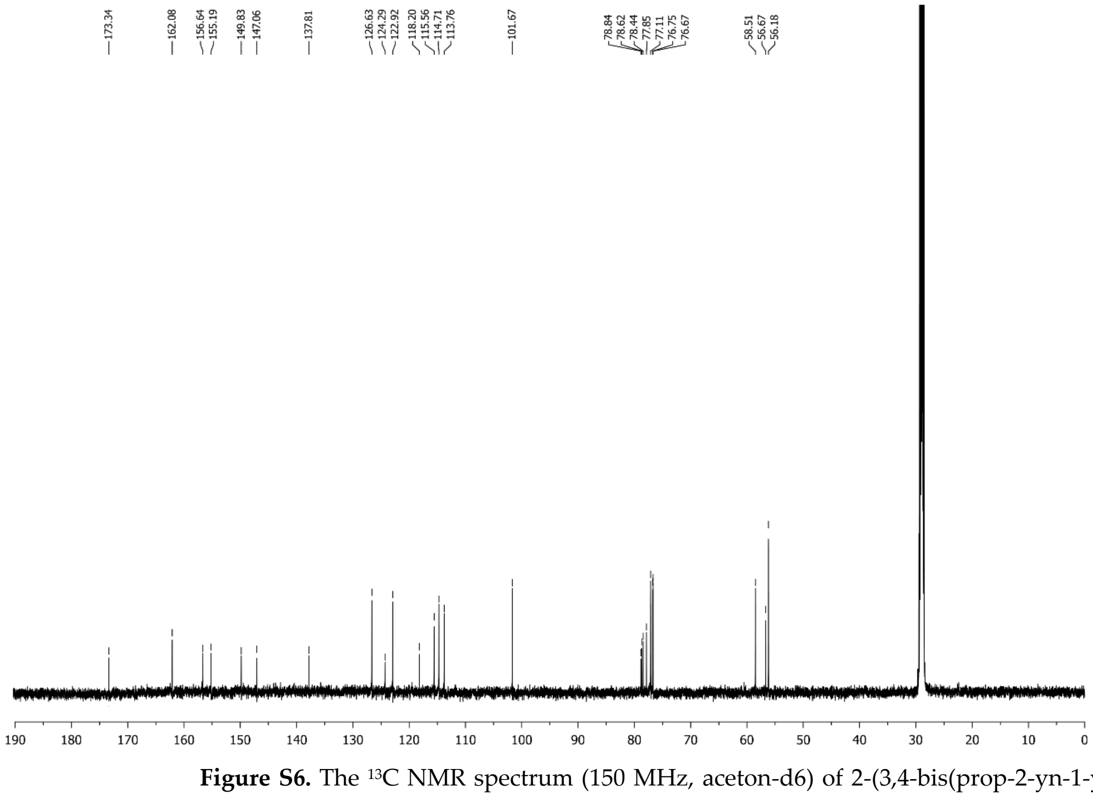

Figure S6. The ^{13}C NMR spectrum (150 MHz, aceton-d6) of 2-(3,4-bis(prop-2-yn-1-yloxy)phenyl)-3,7-bis(prop-2-yn-1-yloxy)- 4H-chromen-4-one **6**.

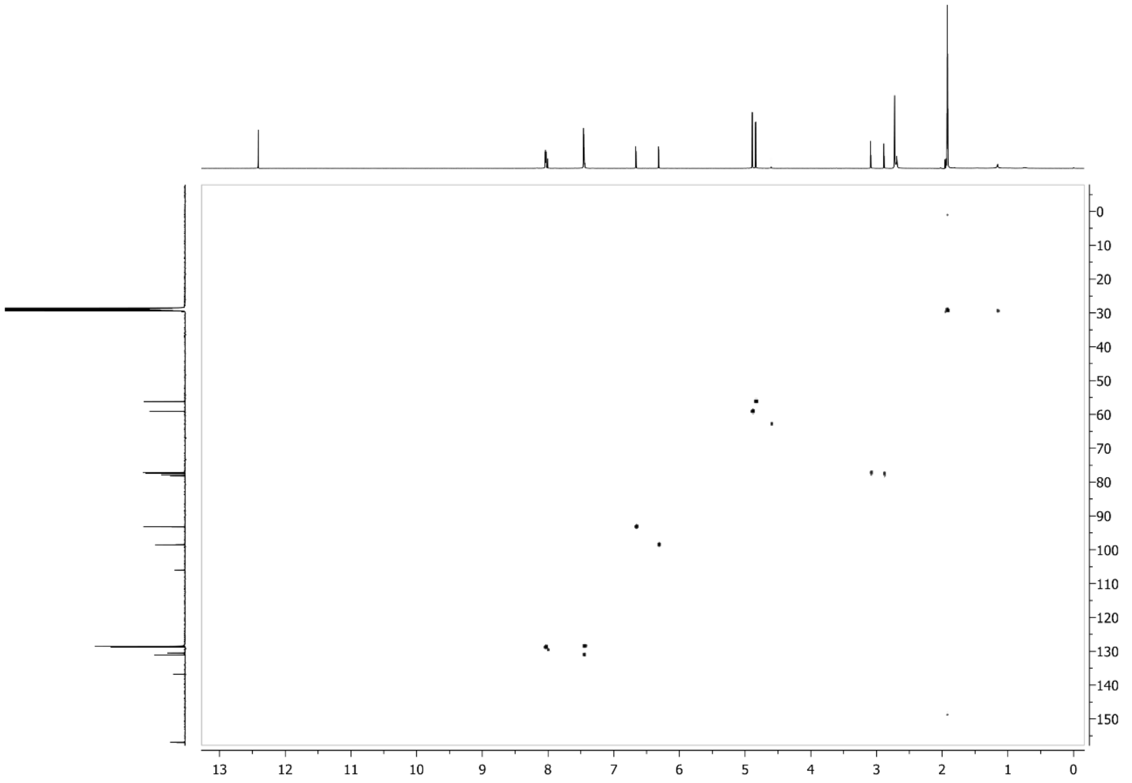

Figure S7. The ^{1}H-^{13}C HSQC spectrum of 5-hydroxy-2-phenyl-3,7-bis(prop-2-yn-1-yloxy)- 4H-chromen-4-one **4**.

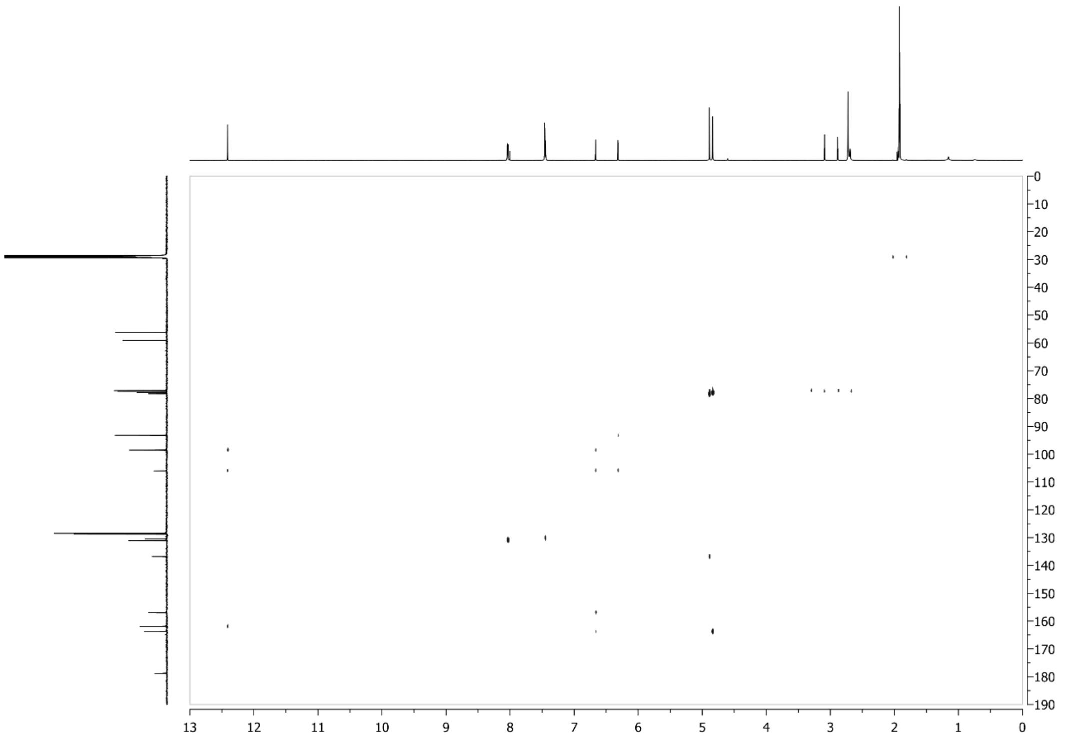

Figure S8. The ^{1}H-^{13}C HMBC spectrum of 5-hydroxy-2-phenyl-3,7-bis(prop-2-yn-1-yloxy)- 4H-chromen-4-one **4**.

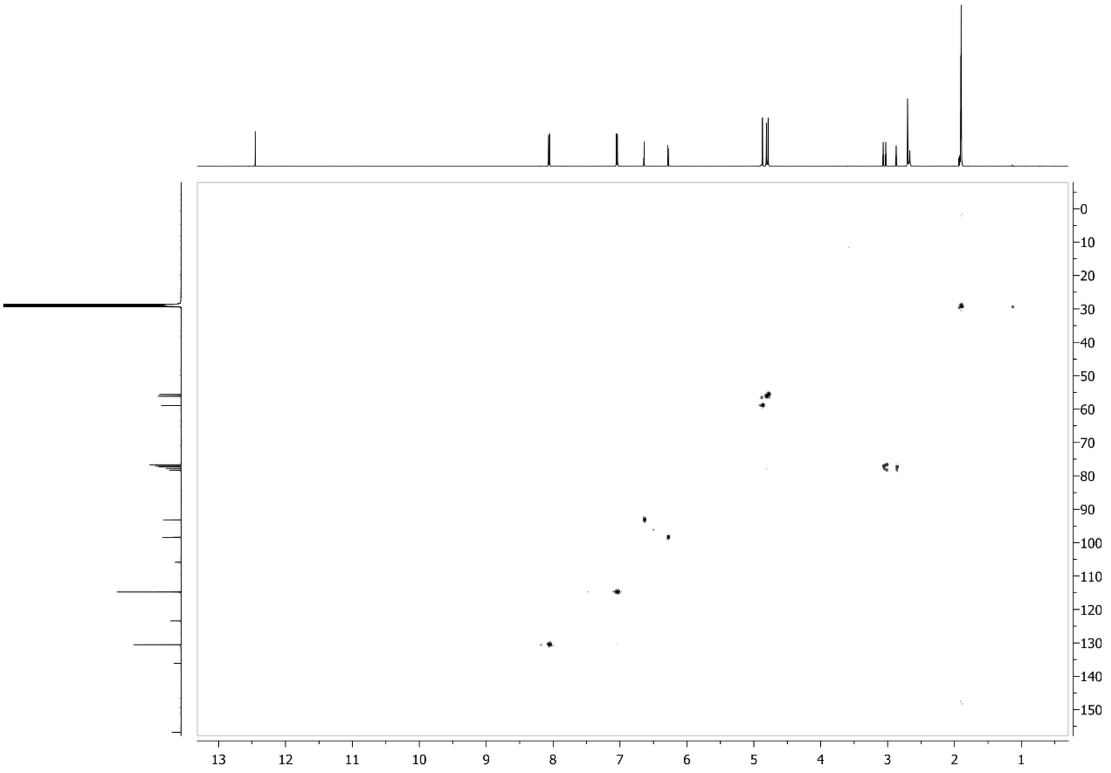

Figure S9. The ^{1}H-^{13}C HSQC spectrum of 5-hydroxy-3,7-bis(prop-2-yn-1-yloxy)-2-(4-(prop-2-yn-1-yloxy)phenyl)-4H-chromen-4-one **5**.

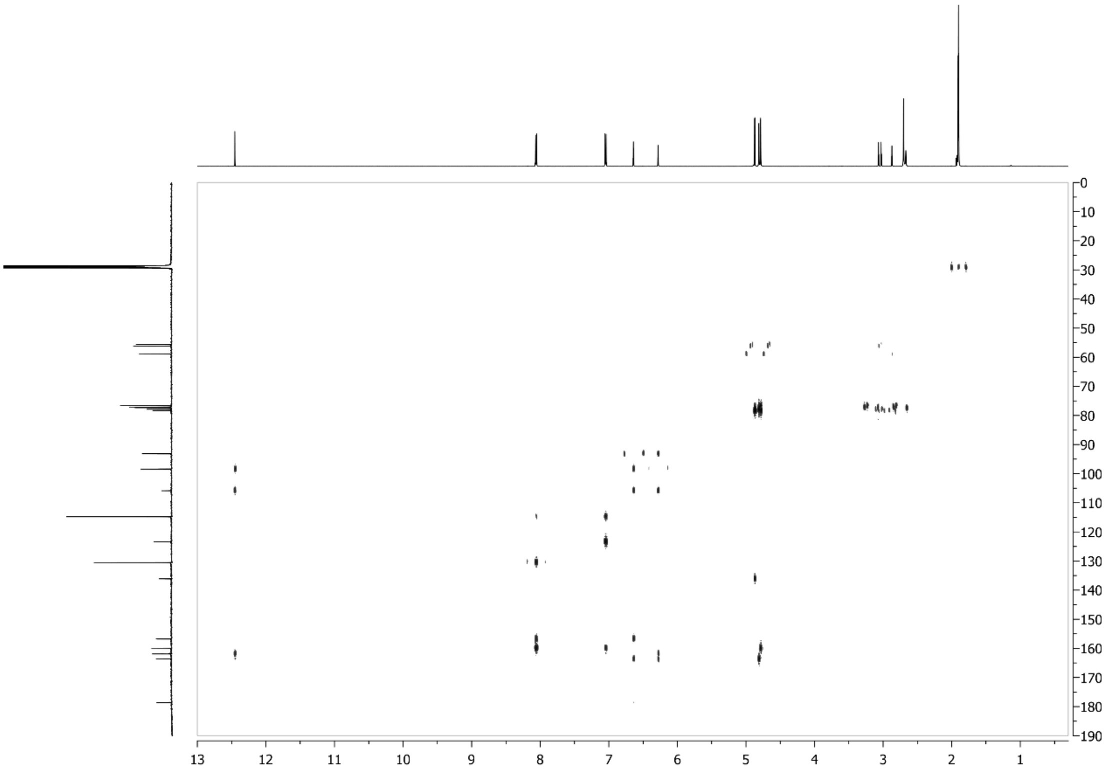

Figure S10. The ^{1}H-^{13}C HMBC spectrum of 5-hydroxy-3,7-bis(prop-2-yn-1-yloxy)-2-(4-(prop-2-yn-1-yloxy)phenyl)-4H-chromen-4-one **5**.
